# Vehicle-related injuries in and around a medium sized Swedish City – bicyclist injuries caused the heaviest burden on the medical sector

**DOI:** 10.1186/s40621-016-0101-8

**Published:** 2017-01-23

**Authors:** Johanna Björnstig, Per-Olof Bylund, Ulf Björnstig

**Affiliations:** Department of Surgery, Umeå University, SE 90187 Umeå, Sweden

**Keywords:** Traffic injuries, Vehicle, Crashes, Bicyclists, Police statistics, Hospitalization

## Abstract

**Background:**

A data acquisition from the medical sector may give one important view of the burden on the society caused by vehicle related injuries. The official police-reported statistics may only reflect a part of all vehicle-related injured seeking medical attention.

The aim is to provide a comprehensive picture of the burden of vehicle related injuries on the medical sector (2013), and to compare with official police-reported statistics and the development year 2000–2013.

**Methods:**

The data set includes 1085 injured from the Injury Data Base at Umeå University Hospital’s catchment area with 148,500 inhabitants in 2013.

**Results:**

Bicyclists were the most frequently injured (54%). One-third had non-minor (MAIS2+) injuries, and bicyclists accounted for 58% of the 1071 hospital bed days for all vehicle-related injuries. Car occupants represented 23% of all injured, and only 9% had MAIS2+ injuries. They accounted for 17% of the hospital bed days. Motorized two wheel vehicle riders represented 11% of the injured and 39% had MAIS2+ injuries and they occupied 11% of the hospital bed days.

Of the 1085 medically treated persons, 767 were injured in public traffic areas, and, therefore, should be included in the official police statistics; however, only a third (232) of them were reported by the police.

The annual injury rate had not changed during 2000–2013 for bicyclists, motor-cycle riders, pedestrians or snowmobile riders. However, for passenger car occupants a decrease was observed after 2008, and for mopedists the injury rate was halved after 2009 when a licensing regulation was introduced.

**Conclusion:**

The Swedish traffic injury reducing strategy Vision Zero, may have contributed to the reduction of injured car occupants and moped riders. The official police-reported statistics was a biased data source for vehicle related injuries and the total number medically treated was in total five times higher. Bicyclists caused the heaviest burden on the medical sector; consequently, they need to be prioritized in future safety work, as recently declared in the Government plan Vision Zero 2.0.

## Background

In just a little over a decade, the number of road fatalities in Sweden has decreased by half according to official statistics. Contributing to this positive trend is the 1997 decision of the Swedish Parliament to adopt the injury reducing strategy called “The Vision Zero” which can be regarded as a road map for the traffic safety work at all levels in the Swedish society. The focus has so far been on reducing fatal injuries, but disabling and non-fatal/serious injuries are also targeted in Vision Zero. The police-reported statistics on fatal injuries has been of acceptable quality; however, when focusing on non-fatal injuries, the official police-reported statistic has weaknesses both in the number of cases and in the categories reported as well as in injury severity classification [1–3]. At the Umeå University Hospital, the only medical facility in the area treating acute injuries, an injury registration of all injuries has been present for several decades. The data from this well-defined catchment area shows the total burden on the medical sector from injuries involving moving vehicles. The data may give essential information that decision makers can use when evaluating areas for preventive actions and when allocating financial resources to mitigate these injuries.

The aim is therefore to provide a comprehensive picture of the etiology, and the burden of injuries on the medical sector, related to moving vehicles. Also by discussing the development during the years 2000–2013, we aim to indicate some factors that may have contributed to the changes. Further, the differences between the official police-reported traffic injury statistics in relation to statistics from the medical sector is analyzed.

## Methods

Our comprehensive data set is based on the injury data of inpatients and outpatients, collected over a one-year period (2013) in Umeå University Hospital’s Injury Data Base (IDB). The IDB includes 10,000 injury cases of all types, treated during one year, at both the hospital and the connected emergency outpatient clinic. These are the only medical facilities treating injuries and they serve a well-defined area of about a 60 km radius around Umeå with 148,500 residents in 2013. Urban Umeå had 110,000 residents, and an additional 38,500 lived in small suburbs and rural areas. Of the total 10,000 persons injured 1085 were related to moving vehicles and were included in the present study. Based on the structure of the health sector in the area, the injured traditionally seek medical attention at the hospital and the associated out-patient clinic. From November through April the area has a winter climate with varying amounts of snowfall and with temperatures down to -20 to -30 °C (-4 to -22 °F) during a couple of weeks in December and January.

The continuous quality control of IDB shows that the level of missed cases from small general practitioner clinics and misses in IDB for those injured in connection to moving vehicles is less than five percent, predominantly persons with very minor injuries. For those hospitalized, the level of missing cases is close to zero, thanks to the compulsory national ICD-10 reporting of the external causes of injuries for all patients with injury diagnoses [4]. Upon arrival to the medical facility, the injured or accompanying persons are asked to fill out a questionnaire about the incident. Ambulance, medical, and police records also provide data to the IDB. Occasionally, an emergency nurse contacts the injured person to fill out missing data. Trained coders process all compiled data into the database. From the IDB, an anonymized data set of 1085 vehicle-related injury cases from the year 2013 was retrieved for analysis.

The severity of the injuries was classified according to the Abbreviated Injury Scale (AIS), where MAIS denotes Maximum AIS, i.e., the AIS-value of the individual’s most serious injury [5]. The AIS scale goes from AIS 1 (minor injury), AIS 2 (moderate), and AIS 3 (serious) to AIS 6 which is a maximal injury-nearly always fatal.

The social-legal context in Sweden and European Union (EU) concerning age, license and vehicle type is the following: To drive a moped (max 25 km/h) or snowmobile, a basic permit is required – the age limit is 15 and 16 respectively. A driver license is since October 2009 required to ride an “EU-moped” with an age limit of 15 years and a maximum speed of 45 km/h. A light motorcycle (<11 kW – 14 US hp) has an age limit of 16, a motorcycle less than <35 kW (47 US hp), has an age limit of 18. For a heavy motorcycle (>35 kW) the age limit is 20–24 years depending on experience. However, organized off-road motorcycle riding such as motocross does not require a license, and there is no age limit. The license age limit for driving passenger cars is 18.

### Statistics

For a trend analysis year 2000 through 2013 for different vehicle categories of the injured, linear regression analysis, has been used. Anova calculation was used for analyzing the three different periods identified for mopedist injuries, 2000–2004, 2005–2009, 2010–2013, related to changes of legal circumstances and vehicle performance. Confidence intervals (CI) for the incidence rates are based on a Poisson assumption of the number of injuries. *P* < 0.05 is the limit for statistical significant changes.

### Ethics

Permission to work with the anonymous data set from the hospital’s injury database was obtained from the Västerbotten County Council’s Research Committee. All analyses have been performed in accordance with the patient’s journal code [6] and the principles of the World Medical Associations Declaration of Helsinki - Ethical Principles for Medical Research Involving Human Subjects (1964) with respect to patient secrecy and confidentiality.

## Results

### Category of road users

#### Medical sector data versus police data

A total of 1085 persons were registered as injured in vehicle crashes and were treated at the hospital and the associated out-patient clinic. Of those, 767 (71%) were injured in public traffic areas, such as public streets/roads, sidewalks, parking lots, etc., which is the selection criterion for being reported in the official police statistics. However, only a third (232; 30%) of those injured in a traffic area were reported by the police. The largest proportion of casualties from traffic known to the police was car occupants, (61% reported), followed by pedestrians hit by a vehicle (59%), but for bicyclists, only 8% of the injured was reported (see Table 1).

**Table 1:** Number and incidence per 1000 residents of injured men and women in different categories and number plus percentage police reported injured on public roads year 2013

Category of vehicle user	Number of injured (col %)	Percentage men/women	Number of injured on public roads	Number reported by the police (col %)	Incidence per 1000 residents
Bicyclist	580 (54%)	52/48	420	33 (8%)	3.9
Car occupant	250 (23%)	46/54	242	148 (61%)	1.7
Motorcyclist	79 (7%)	86/14	19	12 (63%)	0.5
Snowmobile rider	59 (5%)	83/17	4	1 (25%)	0.4
Mopedist	39 (4%)	72/28	32	7 (22%)	0.3
Pedestrian	32 (3%)	53/47	17	19^a^	0.2
Other vehicle occupant	46 (4%)	65/35	33	12 (36%)	0.3
Total	1085 (100%)	56/44	767	232 (30%)	7.3
CI	1020,1150		713,821	202,264	6.9,7.7

^a^2 injured in non-public road but reported by the police

#### Medical sector data

The injury incidence was 7.3 per 1000 residents with a distribution for different vehicle types seen in Table 1. More than half (580; 54%) of the injured seeking medical attention were bicyclists, followed by car occupants (250; 23%) and motorized two wheeled vehicle (MTWV) riders (118; 11%) including both motorcyclists and mopedists. See Table 1.

A trend analysis for injury incidence indicated no significant changes since the year 2000 for bicyclists (*p* = 0.78), motorcyclists (*p* = 0.09), snowmobile riders (*p* = 0.59), or pedestrians (*p* = 0.39). For car occupants a decreasing incidence trend from the year 2009–2013 (0.25 per year, *p* = 0.028) was noted. For mopedists the period could be divided into three parts with significantly different outcomes (*p* = 0.00). In the years of 2000–2004 the injury incidence rates per 100,000 residents were 0.36–0.52, followed by an increase in 2005–2009 (when EU mopeds came to Sweden) with incidence rates of 0.69–0.83. The driver’s license was introduced in October 2009. In the following years of 2010–2013 a major decrease in injured mopedists was found (incidence rates of 0.26–0.44).

#### Fatalities

Six were fatally injured in 2013. Five died on site (consequently not treated at the hospital), and one was treated at the hospital for 38 days. The six fatalities were; two passenger car drivers (head-on collision with another car and a collision with a moose), one van driver (head-on collision with a heavy truck), one snowmobile driver (crashed into a tree), one motorcyclist (crashed into a tree) and one bicyclist (hit by a car).

### Distribution by age and gender

**Fig. 1:**
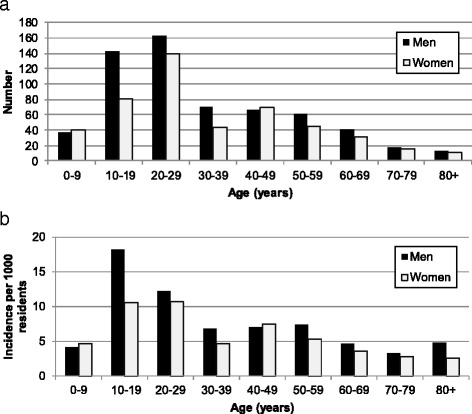
**a** Distribution by age and gender and **b** Incidence of injured vs age and gender per 1000 residents

The distribution of men and women for the 1085 injured was 610 (CI 562,658) (56%) men and 475 (CI 432,518) (44%) women; the male dominance was most pronounced in incidents with MTWV:s and snowmobiles (Table 1). The injury incidence per 1000 residents was 8.2 (CI 7.6,8.8) for men and 6.4 (CI 5.8,7.0) for women and the injury incidence versus age is presented in Fig. 1. More than half (603; 56%) of the injured persons were younger than 30 years (see Fig. 1). Fifty-three (28%) of the 192 drivers of passenger cars were younger than 25 years, and 79 (41%) were younger than 30. For injured bicyclists, car occupants, pedestrians and snowmobile riders the injury frequency peaked in the age group of 20–29. For motorcyclists, the injury frequency peaked in the age group of 10–29 and for mopedists, the frequency was highest in the age group of 10–19.

### Distribution over time

A majority (718;66%) were injured during the warmer part of the year, May through October. Three-quarters (441; 76%) of bicyclist injuries took place during this period, as did those in 92% (108) of the MTWV riders’. However, among car occupants the highest injury frequency was seen during September through March, (177; 71%), as was the case for snowmobile riders as well (58; 98%).

The total injury rate was highest on Saturdays (220; 20%) and between 4 p.m. and 5 p.m. Among bicyclists, the peak day was Saturday (122; 21%), but among injured car occupants, the peak day was Friday (45; 18%). Off-road motorcycle riders were injured mostly during organized practice or competition, and the injury rate peaked during the two weekend days (32; 65%).

### Injury mechanisms

#### Bicyclists

In 419 cases the injured cited external factors as contributing to the crashes and in 122 cases intrinsic personal factors were given, such as the inability to maneuver the bicycle (mostly valid for younger children) (see Table 2). Twenty-nine (5%) collided with four-wheeled motor vehicles and 44 (8%) with other bicyclists. Fifty-three (60%) of the 88 bicyclists, injured between 10 p.m. and 3 a.m., tested positive for, or had visible signs of, being influenced by alcohol.

**Table 2:** Factors contributing to the crash - given by the injured bicyclist

Injury mechanisms – contributing factors	Number (percentage)
Icy road	88 (15%)
Hitting a pothole/crack, object on the road, or contact with curb	45 (8%)
Collision with other 2-wheeled vehicle	44 (8%)
Avoidance maneuver	40 (7%)
Sliding by gravel on road surface	38 (7%)
Fall when getting on/off the bicycle	30 (5%)
Collision with 4-wheeled motor vehicle	29 (5%)
Jumping with the bicycle	21 (4%)
Collision with obstacle	19 (3%)
Sudden stop because of object in a wheel	19 (3%)
Too high speed to handle	16 (3%)
Mechanical failure of the bicycle	14 (2%)
Falling at hard braking	10 (2%)
Fall caused by animal (dog resting)	6 (1%)
Intrinsic factors	122 (21%)
Unknown	39 (7%)
Total	580 (100%)

#### Car occupants

A majority (141; 56%) of the 250 car occupants had collided with other vehicles. Sixty-eight suffered rear-end impacts, and 13 frontal impacts in rear-ending collisions, i.e. a total of 81 were involved in typical queue collisions. Side collisions in intersections caused 32 injury cases, frontal collisions with oncoming vehicles caused 14 cases, and 14 cases were injured in more complicated multiple-impact collisions. Single crashes caused 95 (38%) injury cases, of which at least 27 had crashed into narrow objects, such as trees or light poles. Additionally, 14 (6%) were injured in crashes with wildlife, mostly moose. Among the 195 front-seat occupants 185 (94%) had used seat belts.

#### MTWV riders

A majority of the motorcyclists were injured in off-road riding (56; 71% of the motorcyclists), but 19 (24%) crashed on public roads. In four cases the exact crash site was unknown. Thirty-two (82%) mopedists were injured on public roads. Only five (6%) of all 79 injured motorcyclists collided with other vehicles (two with car, three with other motorcyclists) and the corresponding figure for mopedists was 13 of 39 (33%), of which seven collided with cars and five with other mopeds or an all-terrain vehicle (ATV) and one with a truck.

#### Pedestrians

Twenty-one (66%) of the 32 were hit by passenger cars, three (9%) by bicycles, three (9%) by mopeds and, five (16%) by a bus, truck, or another vehicle.

#### Snowmobile riders

Half (29; 49%) were injured by falls from a snowmobile caused by “irregularities” of the surface, driving into a ditch, jumping, etc., followed by “crashes into immovable objects” (rocks, trees, etc.) (15; 25%). Three (5%) “collided with another snowmobile” while the rest were injured due to a number of other causes.

#### Other vehicle occupants

Of the 46 in this group 26 (57%) were occupants of heavier vehicles such as a bus/coach, truck, or van; 12 (26%) were riders of ATVs and the additional few were occupants of other vehicles.

### Injury severity

About a quarter (295; 27%) sustained non-minor injuries (MAIS 2+) and the number and percentage of cases with MAIS 2+ injuries were (in order of highest percentage first): motorcyclists (34; 43%), bicyclists (193; 33%), mopedists (12; 31%), snowmobile riders (17; 29%), pedestrians (8: 25%), other vehicle occupants (8; 17%), and car occupants (23; 9%).

A trend analysis for 2000–2013 indicated that the percentage of MAIS 2+ injuries has decreased by 1% (*p* = 0.009) per year for bicyclists and 0.5% (*p* = 0.009) per year for passenger car occupants.

### Injury type and localization

The 1085 injured persons sustained a total of 1846 different injuries (Table 3), i.e., 1.7 injuries/injured. The upper extremities, head/face and lower extremities sustained most injuries. Whiplash injury (distortion of the neck) was a common type of injury (215) mostly sustained by car occupants (159; which is 64% of injured car occupants), most frequently from cars sustaining rear-end collisions or side impacts. Bicyclists suffered most of the non-minor injuries (MAIS 2+); they represented 66 (74%) of the 89 persons who suffered concussions plus all 13 of those who had more severe head injuries (AIS 3+) i.e., in total, 79 (77%) of 102 non-minor (AIS 2+) brain injuries. Twelve of the 79 bicyclists with AIS 2+ head injuries had been wearing helmets, 52 did not wear helmets and in 15 cases, the helmet use was unknown. Bicyclists also sustained a large proportion (105, 70%) of the total 150 fractures of the upper extremities and 225 (63%) of all fractures/dislocations. Only two (3%) of 75 snowmobile riders had AIS 2+ head injuries.

**Table 3:** Injury distribution by type and localization - 1846 injuries in 1085 cases

	Head/face	Neck	Thorax/abdomen/pelvis^a^	Upper extremities	Lower extremities	Total
Superficial contusion	120	12	128	146	160	566 (31%)
Distortion	2	215	48	42	54	361 (20%)
Wound	164	3	13	110	118	408 (22%)
Fracture + dislocation	45 + 0	8 + 1	55 + 0	150 + 24	72 + 2	357 (19%)
Concussion + more severe head injury	89 + 13	-	-	-	-	102 (5%)
Other	15	4	15	8	10	52 (3%)
Total	448 (24%)	243 (13%)	259 (14%)	480 (26%)	416 (23%)	1846 (100%)

^a^The category thorax/abdomen/pelvis include injury to the corresponding part of the spine

### Hospitalization

**Fig. 2:**
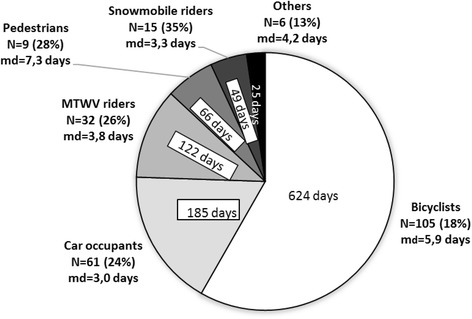
Number of hospital bed days for different categories of vehicle occupants. N = number of hospitalized and percentage of all injured in the category. md = mean days at hospital for the hospitalized

A majority of the injured (857; 79%) were treated in outpatient care. However, 228 (21%) were hospitalized (147 men, 81 women) for a total of 1071 days, i.e., on average 4.7 days/person. Bicyclists occupied 624 (58%) of all hospital bed days, followed by passenger car occupants (185; 17%), and MTWV riders (122; 11%) (see Fig. 2).

Mopedists and pedestrians were the groups with the longest mean times of hospitalization. (7.7 and 7.3 days respectively). Among motorcyclists, one-third (6 of 19) were hospitalized on average for 2.9 days, and of the car occupants one-fourth were hospitalized for an average of 3.0 days.

The person who was hospitalized for the longest time (234 days) was a bicyclist who sustained serious injuries in a single crash.

## Discussion

As defined by the official police-reported statistics, only about 70% of all registered injured, seeking medical attention, were injured in public traffic areas meriting inclusion in the police reported statistics However, only 30% of these cases were, in fact, included in the official statistics. This makes the total number of cases seeking medical attention five times higher than what is shown in the police-reported statistics; a factor that needs to be considered when the overall vehicle related losses to society are estimated. Contributing to this discrepancy is, for example, (i) injuries from crashes on non-public or private areas that are exempted from official statistics, (ii) off-road riding motorcyclists, and (iii) the low reporting frequency (30%) by the police of the cases that nevertheless should have been included. A few other authors have also drawn attention to this problem [1, 2]. Factors influencing police reporting are, for example, the low prioritization of traffic crashes, and the low interest among injured in single crashes to notify the police of their incidents. To improve the situation, the Swedish Traffic Accident Data Acquisition (STRADA), a combined hospital/police registration of traffic injuries, in 2016 was fully introduced at nearly all emergency hospitals (about 65) in Sweden, which is one way to get a more nuanced picture of traffic injury losses and assure a valid injury classification, which the police has difficulties providing. Even so, one-third of all vehicle-related injuries, off-road cases and crashes in non-public areas, usually are not included in STRADA, however with some exemptions as snowmobile and ATV off road crashes. Present data may contribute to a discussion on sharpening definitions for both STRADA and the Vision Zero.

The total number of vehicle-related injuries treated in the medical sector in this geographical area with well-defined demography and control of the data, has decreased for car occupants after 2008 and for riders of EU-mopeds after 2009. For the other road users no significant change could be seen. A reduction is clearly seen in the official traffic death statistics; from 591 (including about 20 suicides) in the year 2000 to 260 (excluding suicides) in 2013. This may reflect the increased awareness among decision makers of the need to take action against the most severe injuries, however non-fatal injuries need more attention. Against the background of an estimated rate of less than five percent missed cases very minor injuries in the area, the presented injury rates are to be regarded as conservative estimates.

### Bicyclists

Bicyclists represented more than half of all persons injured, presented a majority of all non-minor injuries, and occupied 58% of all hospital bed days. The importance of using injury statistics from the medical sector is evident for bicyclists, as only 8% of those who should have been included in the police reported statistics really were xincluded. The injury rate was stable over the years 2000–2013 despite an increased use of bicycle helmets from 14 to 36%, helmet law for children younger than 15 (year 2005), and safer bicycle paths. A slight decrease in the use of hospital beds over the years, may reflect increased helmet use and more frequent use of computer tomography examinations of the brain, thereby reducing the number of head trauma cases hospitalized for clinical observation.

#### Human factor

In the present report, 60% of those injured between 10 p.m. and 3 a.m. had obvious signs of alcohol impairment. Similar results have been shown in Finland and the United Kingdom [7, 8]. In another local study of bicyclists 15 years or older from our area, who suffered concussions or more severe head injuries, we have shown that 80% of those injured during the evening and nighttime had been under the influence of alcohol [9]. Against the background of these facts, the exemption of bicyclists from the Traffic Temperance Law may be discussed. The elderly group (65 and over) was often (34%) treated as inpatients compared with only 16% for those younger than 65. An earlier study from the Umeå area [10] described this elderly group as especially vulnerable, with particular risk for femur neck fractures when getting off a bicycle.

#### Vehicle and equipment

Of the bicyclists, 14% sustained concussions or more severe brain injuries. Of these, two-thirds did not wear helmets. In the state of Victoria in Australia, a bicycle helmet law was introduced in 1990 with a resultant 70% decrease of hospitalized bicyclists with head injuries [11]. Similar data have been presented by other authors [12, 13]. In Sweden, a bicycle helmet law for children younger than 15 was introduced in 2005. In the city of Umeå, it is estimated that 36% of all bicyclists used helmets (among children <10 year of age, 79%) in 2013. A bicycle helmet law for all ages would have great potential to reduce human suffering and the burden on hospital beds for bicyclists. Modern cars also have friendlier fronts that help to limit the trauma energy in the case of collision with bicyclists and pedestrians. One recently introduced system, with the future potential to reduce impact energy, lifts the rear end of the hood and protects the hard structures around the windshield with an airbag. This is of particular importance for bicyclists who have a tendency to impact the front of a car higher than where pedestrians do [14, 15].

#### Environment

Surprisingly, many continue using bicycles in the wintertime in this northern geographical area. Snow/ice is a common factor in the cases where the road surface has been blamed for an injury event [16]. Many people in the Umeå area use studded tires, and in the most recent years, the local traffic authorities have prioritized the snow clearing of walking and bicycle paths with the aim of reducing these injuries. Potholes, cracks, and gravel have caused a number of injuries during summertime, indicating the importance of other general maintenance of bicycle paths. Additionally, over the past several decades, local traffic authorities have recognized and prioritized the importance of separating bicyclists from motorized vehicles to reduce serious and fatal crashes, which is probably one factor for the low crash rate between bicyclists and motor vehicles.

### Passenger car occupants

Passenger car occupants represented less than one-fourth of all injured persons, and the total number has decreased significantly in recent years. Contributing to the decline is probably the broad focus on car occupant safety, both internationally and in the Vision Zero strategy. The decreased number of whiplash injuries is an important factor behind this positive development. The percentage of MAIS2+ injuries was lowest among car occupants, with a decreasing trend after the year 2000. Whiplash injuries, which from an emergency point of view are regarded as minor injuries (AIS 1) have been frequently present, but show a substantial decrease, probably associated with the penetration of better neck restraints in the vehicle fleet and frequent building of roundabouts in recent decades. However, whiplash injuries should not be neglected, as they have a significant risk of causing long-term consequences and medical impairment, see e.g., [17]. Car occupants occupied only 17% of the hospital bed days and the mean time at the hospital was lowest for this group (3.0 days) The police-reporting rate (61%) is in the same range as that reported, for example, in the United States. [18].

#### Human factor

Younger drivers had high injury rates (28% <25 years of age, 41% < 30). The fact that young people have high crash frequency is a well-known fact and is the reason that insurance companies often have higher premiums and deductibles for persons under the age of 25. Factors limiting the crash rate by younger people in Sweden are relatively high licensing age (18 years) and a limited interest among young people to acquire licenses. Only half of those under 20 years of age have licenses.

Alcohol impairment was not routinely tested, but an earlier study from the same hospital showed that a total of 31% of the hospitalized motor vehicle drivers were influenced by alcohol or by other legal or illegal impairing drugs [19].

#### Vehicle and equipment factor

Rear-end crashes were common causing whiplash injuries with sometimes long-term consequences [17, 20]. The introduction of different whiplash injury-reducing restraint systems in modern cars is an important factor behind the fact that the risk of sustaining a whiplash injury with long-term consequences is only half in the best modern passenger cars compared to the older systems [21]. Volvo’s City Safety automatic braking system reduces queue crashes by 25–50% according to a recent US report [22]. When these systems become more widespread in the vehicle fleet, there will probably be a further reduction of rear-end crashes.

The seatbelt usage among front-seat occupants was 94% in the present study. Krafft et al. [23] showed that in modern cars with seatbelt reminders the seatbelt usage rate is about 99%. High seatbelt usage is likely an important factor behind a reduction of MAIS 3+ injuries compared with the past when 38% of those with MAIS 3+ injuries during the 1990’s did not use their seatbelts [24].

#### Environment

The trend of the elimination of red light intersections in favor of roundabouts in recent decades has probably contributed to the reduction of rear-end crashes. Another simple solution that has been tested in the most whiplash prone streets in recent years is doubling the time between red-light intervals, thus reducing the number of abrupt stops. The rear-end crashes on these streets have decreased according to data from local traffic authorities.

Another safety measure has been the introduction of simple mid-barriers on the major rural roads, reducing the severe frontal collisions when compared to earlier data from the same area. These simple mid-barriers have reduced fatal crashes by more than 80% [25]. However, in 2013, three drivers in the present study died in frontal collisions, illustrating the need for more and better mid-barriers.

### Motorcyclists and mopedists (MTWV) riders

The number of injured MTWV riders has varied over the years. This variation maybe depending on factors such as the popularity of different modes of transportation, changes in driver licensing system, and weather conditions. For motorcyclists there was no change 2000–2013 but for mopedists the incidence could be divided into three periods: 2000–2004, 2005–2009 and 2010–2013, representing (i) “the old” moped regulation (max speed 30 km/h) and no formal license and (ii) the import of EU-mopeds (45 km/h), followed by a (iii) driver license requirement starting in October 2009. The injury rate showed a major decrease after the license was introduced.

#### Human factor

Forty-three percent of the motorcyclists had MAIS 2+ injuries, which is the group with the highest percentage of MAIS 2+ injuries. Also, the percentage of injured persons hospitalized was highest among motorcyclists (33%). The average age of injured motorcyclists injured in traffic was 43 and was 26 for those injured off-road. The number of young motorcyclists in traffic has decreased during the last decade, which can be attributed to more complicated driver licensing rules and exceptionally high insurance costs. The 19 motorcyclists injured while riding in traffic did not cause an especially heavy burden for the medical sector. In line with this, Leijdesdorff et al. (2012) [26] showed that light-moped riders have the most severe head injuries, severe trauma, and higher mortality rate compared to motorcyclists. Only two of the 36 mopedists were under the legal age 15, which is a reduction compared to earlier years.

#### Vehicle and equipment factor

Only five of the 79 on + off road driving motorcyclists and only two of the 39 mopedists suffered concussions. Frequent helmet use and the helmet law probably contributed to these low figures. Modern safety features as, for example, anti-lock braking system (ABS) may also reduce the crash risk for motorcyclists [27–29].

#### Environment

Many of the off-road injured persons were injured during organized motocross riding, an activity with an active club in Umeå. Maybe the positive factor is that this activity attracts young people that otherwise may have been driving on public roads. Few of them had serious injuries because of strict regulations on protection gear and safety on the tracks.

The only fatal motorcycle crash was a motorcyclist hitting a tree in the city. Collisions with narrow fixed objects often cause serious and fatal injuries even in low-speed areas. In another study, we showed that two-thirds of all vehicle collisions with light poles with deadly consequences happened in areas with a speed limit under 70 km/h [30].

### Pedestrians

#### Human factor

The total number of injured pedestrians did not decrease in 2000–2013. About 60% were younger than 30. The high proportion of young people probably reflects the demographics of the city, which includes many university students. The use of mobile phones and similar equipment, in particular among the young, may be a contributing factor to inattention among pedestrians according to a number of authors [31–33].

#### Vehicle and equipment

The front-end design of modern cars has probably had an effect on the pattern of injuries. In the Euro-NCAP consumer testing of passenger cars, a pedestrian collision test focused on hood performance is included in the rating. The inclusion of pedestrian collision test may have contributed to improved front-end car design and the previously mentioned airbags offering protection around the window is of value for pedestrians as well [34–36].

#### Environment

During recent decades the local traffic safety work has focused on safe pedestrian and bicycle crossings with the aim of reducing the motor vehicle speed to less than 30 km/h at these points, with aim to reduce serious and fatal pedestrian crashes.

### Snowmobile riders

#### Human

Young men were most frequently injured (83%), given an injury incidence of 0.4 per 1000 residents per year, which is unchanged since year 2000. This is in line with other reports [37, 38].

#### Vehicle and equipment

On January 1^st^, 2016 a helmet law for snowmobile riders was introduced; however, the number of AIS 2+ head injuries was already low (3%) in this group, so the injury reducing effect will likely be minimal.

#### Environment

According to Swedish law snowmobile riding is prohibited on public roads, which contributes to the low frequency of collisions with other vehicles. The one fatality had crashed into a tree. The snowmobile associations fulfill an important role in the injury prevention work by building snowmobile tracks that minimize the risk of colliding with trees and other solid objects.

### Other vehicles

One-third of the injured persons in this group were bus passengers, mostly injured by falling inside of braking, accelerating or colliding buses. Smooth driving would reduce these risks [39].

One of four injured persons in this group was drivers of ATVs which are becoming more popular, especially for off-road driving. One typical injury event was overturning, which in a worst-case scenario can be fatal when one becomes crushed under an overturned vehicle [40, 41].

## Conclusions

Police-reported statistics reflect only a small part of all vehicle-related injuries treated in the medical sector where injured bicyclists had the heaviest impact.

The Swedish government announced, during the fall 2016, “Vision Zero 2.0” with primary focus on unprotected road users, suicides in traffic areas, and use of active crash avoidance systems. The first point seems well motivated against the background of the presented data.

## References

[1] Lujic S, Finch C, Boufous S, Hayen A, Dunsmuir W (2008). How comparable are road traffic crash cases in hospital admissions data and police records? An examination of data linkage rates. Aust N Z J Public Health.

[2] McDonald G, Davie G, Langley J (2009). Validity of police-reported information on injury severity for those hospitalized from motor vehicle traffic crashes. Traffic Inj Prev.

[3] Mattson K, Ungerbäck A. Vägtrafikolyckor. Handledning vid rapportering (in Swedish) [Road Accidents. Guide to reporting]. Borlänge: Swedish Transport Agency; 2013.

[4] The National Board for health and Wellfare (1997). International Statistical Classification of Diseases and related health problems, 10th revision (ICD-10). The National Board for Health and Wellfare.

[5] International Injury Scaling Committee (2008). Abbreviated Injury Scale, 2005, Update 2008.

[6] Patientdatalagen (in Swedish). [Law on Patient Data] lagen.nu/2008:355. Stockholm: Ministry of Social Affairs; 2008

[7] Davidson JA (2005). Epidemiology and outcome of bicycle injuries presenting to an emergency department in the United Kingdom. Eur J Emerg Med.

[8] Airaksinen N, Lüthje P, Nurmi-Lüthje I (2010). Cyclist injuries treated in emergency department (ED): Consequences and costs in South-eastern Finland in an area of 100 000 Inhabitants. Ann Adv Automot Med.

[9] Bylund PO, Björnstig U (2004). Skallskadade cyklister är ofta alkoholpåverkade (in Swedish). [Head injured bicyclists are often under influence of alcohol]. Report 123. Emergency and Disaster Medical Center.

[10] Scheiman S, Moghaddas HS, Björnstig U, Bylund PO, Saveman BI (2010). Bicycle injury events among older adults in Northern Sweden: a 10-year population based study. Accid Anal Prev.

[11] Cameron MH, Vulcan AP, Finch CF, Newstead SV (1994). Mandatory bicycle helmet use following a decade of helmet promotion in Victoria, Australia--an evaluation. Accid Anal Prev.

[12] Lebland J, Beattie T, Culligan C (2002). Effect of legislation on the use of bicycle helmets. CMAJ.

[13] Karkhaneh M, Rowe BH, Saunders LD, Voaklander DC, Hagel BE (2011). Bicycle helmet use four years after the introduction of helmet legislation in Alberta, Canada. Accid Anal Prev.

[14] Fredriksson R, Bylund PO, Oman M (2012). Fatal vehicle-to-bicyclist crashes in Sweden - an in-depth study of injuries and vehicle sources. Ann Adv Automot Med.

[15] Strandroth J, Sternlund S, Lie A, Tingvall C, Rizzi M, Kullgren A (2014). Correlation between Euro NCAP pedestrian test results and injury severity in injury crashes with pedestrians and bicyclists in Sweden. Stapp Car Crash J.

[16] Nyberg P, Björnstig U, Bygren LO (1996). Road characteristics and bicycle accidents. Scand J Soc Med.

[17] Styrke J, Sojka P, Björnstig U, Stålnacke BM (2016). Symptoms, disabilities, and life satisfaction five years after whiplash injuries. Scand J Pain.

[18] NHTSA. The National Automotive Sampling System (NASS) https://www.nhtsa.gov/national-automotive-sampling-system-nass/nass-general-estimates-system. Accessed 29 Nov 2016.

[19] Ahlm K, Björnstig U, Oström M (2009). Alcohol and drugs in fatally and non-fatally injured motor vehicle drivers in northern Sweden. Accid Anal Prev.

[20] Bylund PO, Björnstig U (1998). Sick leave and disability pension among passenger car occupants injured in urban traffic. Spine.

[21] Kullgren A, Krafft M, Lie A, Tingvall C. The effect of whiplash protection system in real life crashes and their correlation to consumer crash test programmes. http://www-nrd.nhtsa.dot.gov/Pdf/ESV/esv20/07-0468-O.pdf. Accessed 29 Nov 2016.

[22] Status Report (2016). Autobrake and forward collision warning are helping drivers avoid a pitfall of travellig congested roads. Status Report.

[23] Krafft M, Kullgren A, Lie A, Tingvall C (2006). The use of seat belts in cars with smart seat belt reminders--results of an observational study. Traffic Inj Prev.

[24] Bylund PO, Bjornstig U (2001). Use of seat belts in cars with different seat belt reminder systems. A study of injured car drivers. Annu Proc Assoc Adv Automot Med.

[25] Carlson A (2009). Uppföljning av mötesfria vägar. [Evaluation of 2 + 1 roads with cable barriers].

[26] Leijdesdorff HA, Siegerink B, Sier CF, Reurings MC, Schipper IB (2012). Injury pattern, injury severity, and mortality in 33,495 hospital-admitted victims of motorized two-wheeled vehicle crashes in The Netherlands. J Trauma Acute Care Surg.

[27] Rizzi M, Strandroth J, Kullgren A, Tingvall C, Fildes B (2015). Effectiveness of motorcycle antilock braking systems (ABS) in reducing crashes, the first cross-national study. Traffic Inj Prev.

[28] Rizzi M, Kullgren A, Tingvall C (2016). The combined benefits of motorcycle antilock braking systems (ABS) in preventing crashes and reducing crash severity. Traffic Inj Prev.

[29] Aikyo Y, Kobayashi Y, Akashi T, Ishiwatari M (2015). Feasibility study of airbag concept applicable to motorcycles without sufficient reaction structure. Traffic Inj Prev.

[30] Björnstig U, Björnstig J (1998). Dödliga singelkrascher i trafiken 1994–95 - med särskild hänsyn till vägens sidoutrymmen (in Swedish). [Fatal single vehicle crashes in traffic 1994–95 with special attention to roadside hazards]. Report 74.

[31] Neider MB, McCarley JS, Crowell JA, Kaczmarski H, Kramer AF (2010). Pedestrians, vehicles, and cell phones. Accid Anal Prev.

[32] Stavrinos D, Byington KW, Schwebel DC (2011). Distracted walking: cell phones increase injury risk for college pedestrians. J Safety Res.

[33] Schwebel DC, Stavrinos D, Byington KW, Davis T, O’Neal EE, de Jong D (2012). Distraction and pedestrian safety: how talking on the phone, texting, and listening to music impact crossing the street. Accid Anal Prev.

[34] Peng Y, Deck C, Yang J, Otte D, Willinger R (2013). A study of adult pedestrian head impact conditions and injury risks in passenger car collisions based on real-world accident data. Traffic Inj Prev.

[35] Zhao H, Yang G, Zhu F, Jin X, Begeman P, Yin Z (2013). An investigation on the head injuries of adult pedestrians by passenger cars in China. Traffic Inj Prev.

[36] Fredriksson R, Dahlgren M, van Schijndel M, de Hair S, van Montfort S (2014). A real-life based evaluation method of deployable vulnerable road user protection systems. Traffic Inj Prev.

[37] Bylund PO, Björnstig U, Enerstam S (1999). Snöskoterrelaterade skadefall vårdade vid Norrlands Universitetssjukhus i Umeå. (in Swedish). [Snowmobile injuries treated at the University Hospital in Umeå] Report 58.

[38] Sy ML, Corden TE (2005). The perils of snowmobiling. Wis Med J.

[39] Björnstig U, Albertsson P, Björnstig J, Bylund PO, Falkmer T, Petzäll J (2005). Injury events among bus and coach occupants - Non-crash injuries as important as crash injuries. J Int Assoc Traffic Saf Sci IATSS Res.

[40] Hall AJ, Bixler D, Helmkamp JC, Kraner JC, Kaplan JA (2009). Fatal all-terrain vehicle crashes: injury types and alcohol use. Am J Prev Med.

[41] Hansson S, Ahlm K, Bylund PO, Eriksson A (2008). All-terrain vehicle fatalities in Sweden, 1992-2004. Scand J Forensic Sci.

